# Epigenetic Genes and Emotional Reactivity to Daily Life Events: A Multi-Step Gene-Environment Interaction Study

**DOI:** 10.1371/journal.pone.0100935

**Published:** 2014-06-26

**Authors:** Ehsan Pishva, Marjan Drukker, Wolfgang Viechtbauer, Jeroen Decoster, Dina Collip, Ruud van Winkel, Marieke Wichers, Nele Jacobs, Evert Thiery, Catherine Derom, Nicole Geschwind, Daniel van den Hove, Tineke Lataster, Inez Myin-Germeys, Jim van Os, Bart P. F. Rutten, Gunter Kenis

**Affiliations:** 1 School for Mental Health and Neuroscience, Department of Psychiatry and Neuropsychology, Maastricht University Medical Centre, Maastricht, The Netherlands; 2 School of Psychology, Open University of the Netherlands, Heerlen, The Netherlands; 3 Department of Neurology, Ghent University Hospital, Ghent, Belgium; 4 Centre of Human Genetics, University Hospital Leuven, and Department of Human Genetics, KU Leuven, Leuven, Belgium; 5 Department of Clinical Psychological Science, Maastricht University, Maastricht, The Netherlands; 6 Department of Psychiatry, and Molecular Psychiatry, Laboratory of Translational Neuroscience, University of Würzburg, Würzburg, Germany; 7 Department of Psychosis Studies, Institute of Psychiatry, King’s Health Partners, King’s College London, London, United Kingdom; Xi’an Jiaotong University School of Medicine, China

## Abstract

Recent human and animal studies suggest that epigenetic mechanisms mediate the impact of environment on development of mental disorders. Therefore, we hypothesized that polymorphisms in epigenetic-regulatory genes impact stress-induced emotional changes. A multi-step, multi-sample gene-environment interaction analysis was conducted to test whether 31 single nucleotide polymorphisms (SNPs) in epigenetic-regulatory genes, i.e. three DNA methyltransferase genes *DNMT1*, *DNMT3A, DNMT3B*, and methylenetetrahydrofolate reductase (*MTHFR*), moderate emotional responses to stressful and pleasant stimuli in daily life as measured by Experience Sampling Methodology (ESM). In the first step, main and interactive effects were tested in a sample of 112 healthy individuals. Significant associations in this discovery sample were then investigated in a population-based sample of 434 individuals for replication. SNPs showing significant effects in both the discovery and replication samples were subsequently tested in three other samples of: (i) 85 unaffected siblings of patients with psychosis, (ii) 110 patients with psychotic disorders, and iii) 126 patients with a history of major depressive disorder. Multilevel linear regression analyses showed no significant association between SNPs and negative affect or positive affect. No SNPs moderated the effect of pleasant stimuli on positive affect. Three SNPs of *DNMT3A* (rs11683424, rs1465764, rs1465825) and 1 SNP of *MTHFR* (rs1801131) moderated the effect of stressful events on negative affect. Only rs11683424 of *DNMT3A* showed consistent directions of effect in the majority of the 5 samples. These data provide the first evidence that emotional responses to daily life stressors may be moderated by genetic variation in the genes involved in the epigenetic machinery.

## Introduction

Levels of emotional reactivity to stressful and pleasant events in daily life reflect an individual’s ability to self-regulate mood states and to cope with environmental challenges. Higher levels of emotional reactivity to daily life stress have been proposed to underlie symptomatology in non-affective psychosis, bipolar disorder, major depressive disorder, and in individuals with familial risk for psychosis [1], [2]. A growing body of evidence from human and animal studies suggests that the ability to regulate emotions is influenced by genetic liability, environmental exposures over the life course, as well as personality traits and coping styles. While the biological basis of emotion regulation and inter-individual variability in the response to stress remains incompletely understood, a flux of recent investigations has shown that epigenetic regulation of gene expression can mediate experience-dependent plasticity (structural and functional changes in the brain in response to environmental exposures and experiences) [3] and may thus play a crucial role in regulating the impact of environmental exposures on psychological and biological functioning of the individual [4]. DNA methylation, that is, the chemical addition of methyl groups to nucleotides, is a key epigenetic mechanism influencing gene expression. The actual transfer of methyl-groups to DNA is mediated by a family of enzymes called DNA methyltransferases (DNMTs). DNMT1 is known to maintain the pre-existing methylation patterns during DNA replication, while DNMT3A and DNMT3B are classified as *de novo* methyltransferases for the establishment of new methylation patterns [5], [6]. The availability of methyl groups is co-determined by the folate-dependent one-carbon pathway with a central role for the enzyme methylenetetrahydrofolate reductase (MTHFR) [7].

Differential responses to stressful stimuli in humans and animals can be programmed by epigenetic mechanisms. Aberrant DNA methylation following adverse experiences has been shown to occur in different stress-related genes [8]. For example, sustained reduction in hippocampal glucocorticoid receptor (GR) expression, accompanied by DNA hypermethylation of the *GR* gene promoter, has been reported in both humans and animals that were prenatally [9] or postnatally exposed to different types of stressors such as maternal separation [10], [11] and childhood maltreatment [12], [13]. Moreover, studies of discordant monozygotic twins with neuropsychiatric disorders consistently show stress-associated alterations in the DNA methylomic profile of the genome [14], [15], [16]. It has also been shown that DNMT-related methylation processes have a crucial role in adult neurogenesis that can mediate experience-dependent plasticity [17]. Although most studies focused solely on the long-term effects of adverse environmental exposures on the methylomic profile of the genome, a recent study has shown that acute psychosocial stress in humans can also induce immediate changes in the DNA methylation status of genes in peripheral blood cells [18]. However, despite rising interest in genes involved in DNA methylation and the role of the epigenetic machinery in mental health and mental disorders, only a limited number of epidemiological studies have examined links between genetic variations in the genes involved in DNA methylation and phenotypes relevant for mental health and psychiatry.

In the current study, a classical gene-environment interaction analysis was applied in order to examine the moderating effect of common single nucleotide polymorphisms (SNPs) in genes relevant for DNA methylation on the association between daily life events and affect. Levels of stressful and pleasant daily life events as well as negative and positive affect were determined using Experience Sampling Methodology (ESM), an innovative method to investigate emotional reactivity to daily life events in an ecologically valid fashion [19]. This method is especially suited to optimize statistical power and sensitivity to detect interplay between genetic variation and environment in humans [19].

Epidemiological findings suggest that psychopathology more likely exists as a continuum rather than a true disease dichotomy [20]. On the other hand, there is a substantial degree of overlap in terms of genetic and environmental risks in patients, relatives of patients and non-patient populations [21]. Moreover, recent findings in a large genome-wide analysis across different psychiatric disorders show that certain SNPs are associated with a range of disorders rather than being disorder-specific [22]. In addition to the pivotal role of replication strategies for genetic and gene-environment interaction studies [23], the present study attempts to discover findings in a group of healthy control subjects and replicate them in a larger sample of general population twins as well as in three other samples of unaffected siblings of psychotic patients, clinical cases of psychosis, and a group of patients with a history of a major depressive disorder currently displaying residual depressive symptoms.

## Methods

### Ethics statement

The Medical Ethics Committee of Maastricht University Medical Centre approved all study procedures, for both healthy participants and patients with mental illness. Written informed consent was obtained from all subjects after they read a document with detailed information about the nature and possible consequences of the study, had verbally discussed any possible concerns with an independent physician who was not involved in the study, and had provided clear indication that they had understood the procedure. For participants under the age of 18, written informed consent was provided by parents or legal guardians. In the Netherlands, patients with mental illness are considered participating citizens who have the right to make independent informed decisions including the autonomous decision to participate in research; therefore consent of relatives was not sought. All patient information was anonymized during the analyses. All researchers at Maastricht University are trained in the guidelines for good clinical practice in research including the practice of obtaining informed consent and recognizing insufficient capacity to provide informed consent, in which case patients are not included in research.

### Samples

We investigated the following five samples, in order to obtain different populations representing a continuum ranging from average psychopathology (general population) to high psychopathology (patient samples):

*Sample I:* Discovery sample, comprising control subjects (n = 112)*; Sample II:* Larger replication sample - female twins from a general population twin study investigating gene–environment interactions in vulnerability for mental disorders (n = 434)*; Sample III:* Unaffected siblings of patients with psychotic disorder (n = 85)*; Sample IV:* Patients with psychotic disorder (n = 110)*; Sample V:* Patients with a history of at least one episode of major depressive disorder, currently displaying residual depressive symptoms (n = 126).

Control subjects in *Sample I* were selected through a random mailing to the residential area of patients and siblings. Inclusion criteria were (i) age 16–55 years old and (ii) sufficient command of Dutch language. Exclusion criteria were (i) use of steroid medication; (ii) current Axis 1 disorder; (iii) lifetime history of psychotic disorder; and (iv) family history of psychotic disorders assessed by the FIGS (NIMH Genetics Initiative, 1992). The subjects from *Sample II* were recruited from the East-Flanders Prospective Twin Survey (EFPTS) [24], a population-based survey that has prospectively recorded all multiple births in the province of East-Flanders since 1964, described in detail elsewhere [25]. The subjects from *Sample III*, healthy siblings of patients with a psychotic disorder, were selected based on the same inclusion and exclusion criteria as described for the control subjects except for the exclusion criterion of family history of psychotic disorder. The subjects from *Sample IV*, patients with a psychotic disorder, were diagnosed following an interview with the positive and negative syndrome scale (PANSS) or operational criteria checklist for psychosis (OPCRIT) [26], [27]. The subjects from *Sample V*, patients with a history of a major depressive disorder, participated in a randomized controlled trial on the effectiveness of mindfulness-based cognitive therapy for patients with residual symptoms of depression [28]. Exclusion criteria for *Sample V* included: fulfilling criteria for a current depressive episode, psychotic episodes in the past year or diagnosis of schizophrenia, and recent (past 4 weeks) or upcoming changes in ongoing psychological or pharmacological treatment.

Control subjects, unaffected siblings, and patients with psychosis *(Samples I, III, and IV)* were selected from three larger cohorts of psychotic patients, siblings, and controls [29], [30] (also see Table S1, and Appendix 1 in Information S1). Participants in the current analysis were classified on the basis of level of psychosis liability and need for care using consistent methodology and criteria. The descriptive characteristics of the participants in the five samples are provided in Table 1.

**Table 1:** Demographic characteristics and ESM variables of the different samples.

	*Sample I*	*Sample II*	*Sample III*	*Sample IV*	*Sample V*
	(N = 112)	(N = 434)	(N = 85)	(N = 110)	(N = 126)
**Sex (M/F)**	(34/78)	(0/434)	(57/28)	(34/76)	(97/29)
**Age, years: (Mean**±**SD)**	33.2±11.5	27.6±7.9	35.9±14.1	34.2±11.4	43.7±9.7
**ESM variables**					
** Stressfulevents (Mean**±**SD)**	0.2±0.2	0.2±0.2	0.2±0.1	0.2±0.3	0.3±0.2
** NA (Mean**±**SD)**	1.2±0.3	1.3±0.3	1.2±0.4	1.7±0.7	2.2±0.7
a **Number of ESM reports**	4716	12481	3600	4488	5887
** Pleasant events (Mean**±**SD)**	1.8±0.5	1.5±0.6	1.8±0.5	1.7±0.6	1.9±0.4
** PA (Mean**±**SD)**	5.0±0.6	4.7±0.7	5.0±0.7	4.5±0.9	4.0±0.7
b **Number of ESM reports**	4219	11025	3263	3955	4913

Sample I = Healthy control; Sample II = Twins from the general population; Sample III = Unaffected siblings of psychotic patients; Sample IV = Patients with psychotic disorder; Sample V = Patients with a history of a major depressive disorder currently displaying residual depressive symptoms.

a Number of ESM reports when scores for both stressful event and NA are available,

b number of ESM report when scores for both pleasant event and PA are available.

### Experience Sampling Method (ESM)

ESM is a random time-sampling self-assessment technique. Subjects received a digital wristwatch that emitted a signal 10 times a day on 6 consecutive days, at unpredictable moments between 07∶30 and 22∶30. After each ‘beep’, subjects completed ESM self-assessment forms concerning current context, thoughts, emotions, and psychotic experiences. Subjects were instructed to complete their reports immediately after the beep, thus minimizing memory distortions. Reports were considered valid when subjects responded within 15 min after the beep, as determined by comparing the actual beep time with the reported time of completion. In line with previous reports, a valid response to at least one–third of the emitted beeps was required for inclusion in the analyses [31], [32]. Several studies have demonstrated the feasibility, validity, and reliability of ESM in general populations as well as in patient populations [19], [25], [32], [33].

### Daily stressful and daily pleasant events

After each beep, the most important event that had occurred between the current and the previous beep was reported. This event was subsequently rated on a 7-point Likert scale (–3 = very unpleasant, 0 = neutral, 3 = very pleasant).

A “stressful event” was conceptualized as the subjectively appraised stressfulness of preceding events [1]. Responses were recoded to allow high scores to reflect stress (–3 = very pleasant, 0 = neutral, 3 = very unpleasant). Thus, the positive scores (1, 2, and 3) were considered as stressful events, with the higher scores in this variable reflecting higher levels of experienced stress.

A “pleasant event” was conceptualized as the subjectively appraised pleasantness of distinct events [34]. In the recoded Likert scale for daily events, pleasant events were defined as negative scores and −3 in this variable reflects the most pleasant events.

### Negative and Positive Affect

After each beep, participants were also asked to answer questions regarding their mood state on 7-point Likert scales (1 = not at all, 7 = very). Negative Affect (NA) was defined as the mean score on five adjectives related to negative mood states (feeling down, guilty, insecure, lonely, and anxious) at that moment. Similarly, Positive Affect (PA) was defined as the mean score on positive mood-related adjectives (feeling relaxed, satisfied, and cheerful).

### Selection of genes, SNPs, and genotyping procedures

Four genes with evident roles in DNA methylation processes in the brain were selected: *DNMT1*, *DNMT3A*, *DNMT3B*, and *MTHFR*. For *MTHFR*, two known functional SNPs, an A-to-C substitution at nucleotide 1298 (rs1801131) and a C-to-T substitution at nucleotide 677 (rs1801133), were selected based on previous reports of their associations with mental disorders [35]. Currently neither functional polymorphisms, nor associations have been described for the *DNMT* family genes. Therefore, thirty-one tagging SNPs in the *DNMT* family genes (*DNMT1, DNMT3A*, and *DNMT3B*) were captured by the Haploview add-on software package SNAGGER [36] with the following settings: minor allele frequency>5%; pairwise *r* ^2^>0.8; and distance from closest SNP>60 bp. Linkage disequilibrium blocks were determined using data from the Hap Map project (www.hapmap.org).

DNA was extracted from blood, saliva, or buccal epithelium for different samples. The selected SNPs were determined by Sequenom MassARRAY iPLEX platform at the facilities of the manufacturer (Hamburg, Germany). Of the 33 SNPs originally included (see Table S2 in Information S1), one SNP (rs13427202 of *DNMT3A*) was excluded because of Hardy–Weinberg disequilibrium (*p*<.001) and another SNP (rs709046 of *DNMT3B*) was excluded from analyses because it showed no variation. Thus, a final set of 31 SNPs in 4 genes was suitable for further analysis.

### Statistical analysis

All analyses were performed using STATA 12.1 (StataCorp. 2011). ESM data have a hierarchical structure. Multilevel linear regression analyses (using the xtmixed command) are ideally suited for multiple measurements of affect (level 1) that are clustered within subjects (level 2), some of whom in turn were clustered within twin pairs (level 3). The regression coefficients of the multilevel linear regression models reflect the (estimated) influence of a particular predictor on the outcome of interest. The dominant genetic model for the SNPs was the best fitting model based on Akaike’s Information Criterion (AIC), and therefore the SNPs were coded as 0 (AA) and 1 (Aa or aa).

First, main effects were analyzed, that is, i) the association between momentary stressful events and NA, and between momentary pleasant events and PA (testing main effect of daily events on affect), ii) the association between the 31 SNPs and NA or PA (testing genetic main effects on affect), iii) the association between the 31 SNPs on the one hand and momentary stressful events and momentary pleasant events on the other (testing gene-environment correlations). These analyses were performed in all five samples.

Second, a multi-step approach was applied to test whether the association between daily events and affect would differ as a function of the SNPs. Two-way interaction terms between the individual SNPs and stressful events were included when analyzing NA, and between individual SNPs and pleasant events when analyzing PA. In the first step, we tested whether the association between daily events and affect was moderated by the SNPs in *Sample I*. In step two, those two-way interaction terms that showed statistical significance after applying the multiple testing correction in *Sample I* were analyzed in *Sample II.* The effective number of tests (considering correlation between multiple loci within a single gene) was estimated in this study using the method of Li and Ji [37], yielding a value of 20. Therefore, instead of using a Bonferroni-corrected alpha level of α/31, we used α/20 (α<0.0025), which is less conservative. Finally, only SNPs showing statistically significant interactions in both *Sample I* and *Sample II* were subsequently examined in the three remaining samples (*Sample III–V*). All analyses were a priori adjusted for age and sex (with exception of the exclusively female twin sample).

## Results

### Main effect analysis

All five samples showed significant associations between stressful events and negative affect on the one hand, and between pleasant events and positive affect on the other, independently of genotype (Table 2). In *Sample V,* the association between emotional reactivity and stressful and pleasant events was significantly stronger than in the discovery *sample I* (*p*<0.0001).

**Table 2:** Associations between daily stressful life events and NA, and between pleasant life events and PA.

	*Sample I*	*Sample II*	*Sample III*	*Sample IV*	*Sample V*
**Stressful event and NA**	a0.1487	0.1660	0.1535	0.1667	0.2801
	b(0.1292, 0.1682)	(0.1525, 0.1796)	(0.1304, 0.1765)	(0.1390, 0.1943)	(0.2543, 0.3059)
	*P = 0.000*	*P = 0.000*	*P = 0.000*	*P = 0.000*	*P = 0.000*
**Pleasant event and PA**	0.1713	0.1682	0.1503	0.1864	0.3430
	(0.1456, 0.1969)	(0.1516, 0.1849)	(0.1202, 0.1805)	(0.1553, 0.2176)	(0.3100, 0.3760)
	*P = 0.000*	*P = 0.000*	*P = 0.000*	*P = 0.000*	*P = 0.000*

Sample I = Healthy control; Sample II = Twins from the general population; Sample III = Unaffected siblings of psychotic patients; Sample IV = Patients with psychotic disorder; Sample V = Patients with a history of a major depressive disorder currently displaying residual depressive symptoms.

a Regression coefficient,

b 95% confidence interval.

Main effects of SNPs were not significant, neither for NA, nor for PA. No effects of SNPs on either stressful or pleasant events were observed, ruling out gene-environment correlation and indicating independence of genetic and environmental risks.

### Gene-environment interactions

#### SNPs× Daily stressful events on NA

*Step 1:* Interactive effects between 31 SNPs and stressful events on NA were tested in *Sample I*. Significant SNP × stressful events interactions were found in five SNPs after correction for multiple testing: three SNPs of *DNMT3A* (rs11683424, rs1465764, and rs1465825), one SNP of *DNMT3B* (rs2424913), and one SNP of *MTHFR* (rs1801131). *Step 2:* Among the five SNPs discovered in *Sample I*, three SNPs of *DNMT3A* (rs11683424, rs1465764, and rs1465825), and the SNP of *MTHFR* (rs1801131) were confirmed in the larger replication *Sample II* (Table 3). *Step 3:* SNPs showing statistically significant interactions in both *Sample I* and *Sample II* were examined in the three remaining samples. Three SNPs of *DNMT3A* (rs11683424, rs1465764, and rs1465825) showed a significant association in at least one of these three samples (Table 3).

**Table 3:** Two-way interaction analyses between SNPs and daily stressful life events in models of NA.

		Step 1	Step 2	Step 3		
SNP(Gene)		*Sample I*	*Sample II*	*Sample III*	*Sample IV*	*Sample V*
**rs11683424(** ***DNMT3A*** **)**	**CC**	a0.1727 b(82)	0.1762 (312)	0.1501 (62)	0.1807 (73)	0.3008 (78)
		c(0.1502, 0.1953)	(0.1606, 0.1918)	(0.1238, 0.1764)	(0.1454, 0.2160)	(0.2678, 0.3338)
	**CT/TT**	0.0773 (30)	0.1355 (116)	0.1653 (23)	0.1442 (37)	0.2475 (48)
		(0.0384, 0.1161)	(0.1072, 0.1637)	(0.1175, 0.2131)	(0.0996, 0.1887)	(0.2061, 0.2888)
		d *P = 0.0000*	*P = 0.0133*	*P = 0.5848*	*P = 0.2075*	*P = 0.0482*
**rs1465764(** ***DNMT3A*** **)**	**AA**	0.1384 (99)	0.1758 (367)	0.1675 (72)	0.1711 (95)	0.2734 (109)
		(0.1178, 0.1590)	(0.1612, 0.1904)	(0.1429, 0.1921)	(0.1422, 0.2000)	(0.2458, 0.3011)
	**AT/TT**	0.2374 (13)	0.1023 (62)	0.0534 (13)	0.1109 (14)	0.3250 (17)
		(0.1771, 0.2975)	(0.0650, 0.1396)	(–0.0123, 0.1191)	(0.0100, 0.2117)	(0.2536, 0.3963)
		*P = 0.0022*	*P = 0.0003*	*P = 0.0014*	*P = 0.2602*	*P = 0.1863*
**rs1465825(** ***DNMT3A*** **)**	**TT**	0.1238 (65)	0.1811 (263)	0.1671 (51)	0.1576 (54)	0.3040 (74)
		(0.0992, 0.1483)	(0.1634, 0.1988)	(0.1366, 0.1976)	(0.1173, 0.1978)	(0.2693, 0.3386)
	**TC/CC**	0.1912 (47)	0.1447 (166)	0.1356 (34)	0.1749 (56)	0.2505 (52)
		(0.1592, 0.2232)	(0.1235, 0.1658)	(0.1004, 0.1708)	(0.1367, 0.2130)	(0.2118, 0.2891)
		*P = 0.0010*	*P = 0.0097*	*P = 0.1851*	*P = 0.5410*	*P = 0.0433*
**rs2424913(** ***DNMT3B*** **)**	**CC**	0.1955 (47)	0.1564 (142)			
		(0.1629, 0.2281)	(0.1335, 0.1794)			
	**CT/TT**	0.1227 (65)	0.1736 (275)			
		(0.0983, 0.1469)	(0.1565, 0.1907)			
		*P = 0.0004*	*P = 0.2404*			
**rs1801131(** ***MTHFR)***	**AA**	0.1727 (64)	0.1814 (211)	0.1374 (39)	0.1831 (54)	0.2847 (64)
		(0.1502, 0.1953)	(0.1622, 0.2006)	(0.1008, 0.1740)	(0.1414, 0.2247)	(0.2486, 0.3207)
	**AC/CC**	0.1142 (48)	0.1355 (216)	0.1642 (46)	0.1559 (55)	0.2756 (62)
		(0.0857, 0.1427)	(0.1331, 0.1715)	(0.1344, 0.1938)	(0.1185, 0.1932)	(0.2387, 0.3125)
		*P = 0.0010*	*P = 0.0360*	*P = 0.2650*	*P = 0.3401*	*P = 0.7314*

Sample I = Healthy control; Sample II = Twins from the general population; Sample III = Unaffected siblings of psychotic patients; Sample IV = Patients with psychotic disorder; Sample V = Patients with a history of a major depressive disorder currently displaying residual depressive symptoms.

a Regression coefficient indicates changes in negative affect associated with changes in subjectively appraised stress,

b number of subject per genotype,

c 95% confidence interval,

d P-value for the interactive effect model.

#### SNPs× Daily pleasant events on PA

A similar stepwise approach was applied to examine the interactive effects between 31 SNPs on the one hand, and pleasant events on the other, in models of PA. Only one *DNMT1* SNP (rs6511677) showed a significant SNP × pleasant event interaction in the discovery *Sample I,* withstanding multiple testing correction (B = 0.1135; CI 95%: [0.05620–0.17080]; *p = 0.0001*). This effect did not remain significant in the replication *Sample II*, and therefore we did not proceed with *Step 3* of the analyses.

## Discussion

**Figure 1:**
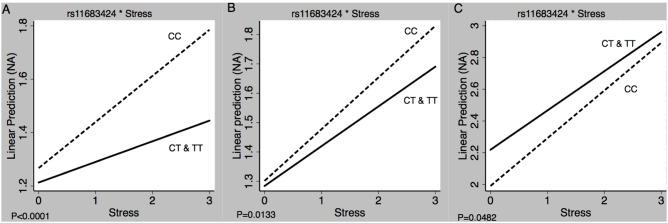
Results of multilevel regression analyses estimating the association between daily stressful life events and NA, as a function of SNP rs11683424 in the *DNMT3A* gene. The buffering effects were consistently observed in individuals carrying the T allele in three samples of (A) healthy control subjects (B) female twins from the general population, and (C) patients with a history of at least one episode of major depressive disorder, currently having residual depressive symptoms.

Our results show that stressful and pleasant daily life events predict immediate emotional changes in different study groups, in accordance with previous clinical and epidemiological reports [1]. From the perspective of G×E interaction, our results show, for the first time, that genetic variability in the *DNMT* gene family may moderate the effect of daily stressful events on negative affect in a different range of psychiatric disorders. Significant interaction that was directionally similar and consistent in three independent samples was observed for only 1 SNP (rs11683424 in the *DNMT3A* gene). Our findings suggest that T-allele carriers (CT or TT) of rs11683424 can buffer the impact of daily stressful events on negative affect – an effect that was observed in two independent samples of healthy subjects and in a sample of patients with residual symptoms of depression (Figure 1). The so-called ‘flip-flop’ associations (i.e., opposite alleles conferring risk [38]) were found in the other significant SNPs across different samples (Table 2).

DNA methylation is known to be involved in the regulation of stress-related gene expression and the DNMT3A enzyme is widely expressed in brain circuitry mediating emotional processing and regulation [39]. It has been shown that increased expression of DNMT3A in the nucleus accumbens (NAc) during subchronic stress in mice induces depressive-like behavior, while it negatively regulates cocaine reward [39]. Interestingly, inhibition of the DNMT family enzymes has been shown to produce antidepressant effects and to reverse the depressive-like behavior in mice. Furthermore, increased expression of DNMT3A in NAc induced by chronic stress was accompanied by induction of dendritic spines in NAc neurons which is known to underlie differential emotional responses [39].

This suggests that the DNMT3A enzyme thus regulates emotional behavior, which is in line with our observations that responses to stressful stimuli may vary between individuals who carry various polymorphisms in the *DNMT3A* gene.

**Figure 2:**
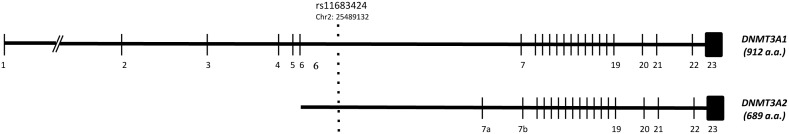
A schematic overview of two major protein-coding transcripts of the *DNMT3A* gene. The numbered blocks are exons, the connecting lines introns. Genetic variability in *DNMT3A* mainly occurs in intronic regions while the exonic regions are very well conserved. The dashed line indicates the location of the tagSNP rs11683424, which we found to significantly moderate the association between daily stressful events and negative affect. The *DNMT3A2* transcript seems to be regulated by an alternative promoter upstream of exon 7, and bears a unique untranslated exon 4 kb upstream of exon 7 (termed exon 7a). rs11683424 is situated upstream of the *DNMT3A2* transcription start site. Gene structure and names of the protein coding transcripts were derived from the Ensembl database (www.ensembl.org).

The *DNMT3A* gene harbors 23 exons, resulting in various possible protein-coding transcripts. Two transcripts code for the full length DNMT3A1 protein (exons 2–23), and one transcript for a shorter DNMT3A2 isoform. The latter transcript contains exons 7–23 and the protein lacks the N-terminal 223 amino acid residues of the full-length DNMT3A protein [40], [41]. Expression of the *DNMT3A2* transcript seems to be regulated by an alternative promoter, upstream an additional unique untranslated exon located 4 kb upstream of exon 7 [40]. SNP rs11683424 is located in intron 6, approximately 14 kb upstream of this extra exon for the *DNMT3A2* transcript and may therefore reside in a regulatory region for *DNMA3A2* expression (Figure 2).

Furthermore, it is possible that rs11683424 variation is associated with alternative splicing of the *DNMT3A* gene. According to the Ensembl 2014 database [42], variation in rs11683424 may result in nonsense-mediated decay in a transcript of *DNMT3A*. However, in vitro studies are needed to determine the splicing role of rs11683424 in different transcripts of the *DNMT3A* gene.

Taken together, it is tempting to speculate that variation in rs11683424 has a functional impact on the expression of *DNMT3A2* and that, as such, this genetic altered methylation activity is related to negative emotional experiences after daily stressful events.

Our results suggest that *DNMT3A* genetic variation impacts on immediate emotional responses to daily stressful events. This finding is in concert with recent work that shows a dynamic regulation of DNA methylation in the oxytocin receptor gene (of which a major role in emotion regulation has been widely reported) after acute psychosocial stress [18]. On the other hand, these findings may also reflect a role for genetic variation in the *DNMT3A* gene in life-long individual differences in epigenetic programming of emotion regulation [43]. Emotion regulation strategies such as situation selection, situation modification, attentional deployment, cognitive reappraisal, and response modulation [44] can be acquired by learning processes. Recent research indicates that synaptic plasticity underlying learning and memory can be shaped by dynamic epigenetic changes during critical periods of brain development [45].

To the best of our knowledge, this is the first gene-environment interaction study investigating the inter-individual variation in epigenetic regulatory candidate genes at the fixed genomic level in mental disorders. In the present study, we utilized several analytic strategies to avoid major challenges in the study of gene-environment interaction studies. First of all, we designed a case-only analysis, which is proposed as a more powerful method to identify loci involved in gene-environment interactions [46]. Second, the high failure rate in replication attempts in gene-environment interaction studies have been ascribed to inconsistent measurements of environmental factors and phenotypes, and inappropriate study designs [23]. In this study, we assured consistency in design, measurement of environmental factors, and phenotype assessment across the five samples. Furthermore we applied ESM for the assessment of emotional reactivity to stressors occurring in the flow of daily life. This method allows insight into the course of emotional responses and the influence of genes and environmental stimuli in a momentary, ‘real-world’ design by providing a prospective collection of cumulative, repeated measures of proximal environmental risk factors [19], [47]. Moreover, ESM enables researchers to collect all environmental and phenotypical data prospectively and in a longitudinal manner for different samples to reduce the risk of false positive reports in replication studies.

### Limitations

Our study has some limitations that should be noted. First, in the second replication sample of twins from the general population, only female participants were included. Second, despite the fact that the ESM method increases the total number of observations (Table 1) and enhances the power of analyses, the sample size in some of our study groups was rather small. However, the current study is – to the best of our knowledge – the largest study to date on genetic moderation of the effect of daily life stimuli on positive and negative affect, while minimizing Type I errors by using a multi-step replication strategy.

Further, we observed differences in the direction of association for some SNPs in the different population samples. Such ‘flip-flop’ patterns of association are a relatively common phenomenon seen in many genome-wide association studies [48] and might be related to the assessment of single-locus associations in the presence of multi locus effects in a complex disorder [38], [49]. Given that emotional responses to stimuli in daily life are very likely determined by complex interactions of a plethora of genetic and environmental factors occurring throughout the individual’s life span, studying the impact of a single environmental factor in interaction with a few selected genes (here: 4 genes out of a approximate total of 30,000 genes) of course represents a very major challenge.

## Conclusion

The present findings suggest that genetic variation in one of the main DNA methylation regulatory enzymes, DNMT3A, moderates emotional responses to daily life stressors. The location of SNP rs11683424 in a putative regulatory region of the alternative *DNMT3A2* transcript tempts us to speculate that this variant may influence the expression of DNMT3A2, the short length isoform of the enzyme, specifically. Genetic altered activity of methylation processes may shape emotional responses to daily life stressors in adulthood. Further studies are required in order to elucidate this, and to further clarify the role of DNMT3A and its isoforms, in the neurobiological underpinning of emotion regulation.

## Supporting Information

Information S1 **Appendix 1**, Descriptions of cohort study No. 2 mentioned in Table S1. **Table S1**, An overview of the pooled ESM studies for sample I, III and IV, participant status and references to original study descriptions. **Table S2**, List of tagging and functional SNPs.
(ZIP)
