# Supplementary material for: Epigenetic Genes and Emotional Reactivity to Daily Life Events: A Multi-Step Gene-Environment Interaction Study
Source: PLoS One. 2014 Jun 26;9(6):e100935. doi: 10.1371/journal.pone.0100935 (PMC4072714; doi:10.1371/journal.pone.0100935)
Supplement: Information S1 — Appendix 1, Descriptions of cohort study No. 2 mentioned in Table S1. Table S1, An overview of the pooled ESM studies for sample I, III and IV, participant status and references to original study descriptions. Table S2, List of tagging and functional SNPs. (ZIP) [file pone.0100935.s001.zip › Supporting information S1/Appendix 1.docx]

**Appendix 1. Descriptions of cohort study No. 2 mentioned in Supp. Table 1.**

The sample consisted of 44 patients with non-affective psychotic disorder, 48 individuals with genetic risk for psychosis (first-degree relatives of individuals with psychotic disorder), and 49 healthy controls. Inclusion criteria were (i) age 18-65 years; (ii) sufficient command of the Dutch language to understand instructions and give informed consent. Exclusion criteria were (i) intellectual impairment (IQ<80); (ii) head trauma or neurological disorder; (iii) endocrine disorder; (iv) pregnancy or lactation (women only); (v) current or previous use of illicit drugs; (vi) use of alcohol in excess of five standard units per day. An additional exclusion criterium for subjects with genetic risk for psychosis or controls was present or history of psychiatric illness according to the explicit diagnostic criteria of the DSM-IV-TR ([APA, 2000](#_ENREF_1)). The standing medical ethics committee approved the study; subjects signed informed consent after description of the study. Subjects were recruited through pamphlets, advertisements in local newspapers, and random mailing procedures in the local area. Subjects with genetic risk for psychosis were additionally recruited by contacting local family organizations for relatives of patients with a psychotic disorder.
