# Supplementary material for: Epigenetic Genes and Emotional Reactivity to Daily Life Events: A Multi-Step Gene-Environment Interaction Study
Source: PLoS One. 2014 Jun 26;9(6):e100935. doi: 10.1371/journal.pone.0100935 (PMC4072714; doi:10.1371/journal.pone.0100935)
Supplement: Information S1 — Appendix 1, Descriptions of cohort study No. 2 mentioned in Table S1. Table S1, An overview of the pooled ESM studies for sample I, III and IV, participant status and references to original study descriptions. Table S2, List of tagging and functional SNPs. (ZIP) [file pone.0100935.s001.zip › Supporting information S1/Table S1.docx]

| **Study** | **General status** | **(N)** | **Reference** |
| --- | --- | --- | --- |
| Cohort 1 | Healthy controls | 76 | [[28](#_ENREF_1)] |
| Cohort 2 | Healthy controls | 36 | See appendix 1 |
|  | Relatives of patients with Psychosis | 25 |  |
|  | Psychosis | 22 |  |
| Cohort 3 | Relatives of patients with Psychosis | 60 | [[29](#_ENREF_2)] |
|  | Psychosis | 88 |  |

**Table S1.** **An overview of the pooled ESM studies for sample I, III and IV, participant status and references to original study descriptions**
